# Functional Analysis of a Carboxylesterase Gene Associated With Isoprocarb and Cyhalothrin Resistance in *Rhopalosiphum padi* (L.)

**DOI:** 10.3389/fphys.2018.00992

**Published:** 2018-07-25

**Authors:** Kang Wang, Yanna Huang, Xinyu Li, Maohua Chen

**Affiliations:** State Key Laboratory of Crop Stress Biology for Arid Areas, Key Laboratory of Crop Pest Integrated Pest Management on the Loess Plateau of Ministry of Agriculture, Northwest A&F University, Yangling, China

**Keywords:** carboxylesterase, *Rhopalosiphum padi*, isoprocarb, pyrethroid, metabolism resistance

## Abstract

Carboxylesterase (CarE) is an important class of detoxification enzymes involved in insecticide resistance. However, the molecular mechanism of CarE-mediated insecticide resistance in *Rhopalosiphum padi*, a problematic agricultural pest, remains largely unknown. In the present study, an isoprocarb-resistant (IS-R) strain and a cyhalothrin-resistant (CY-R) strain were successively selected from a susceptible (SS) strain of *R. padi*. The enzyme activity indicated that enhanced carboxylesterase activity contributes to isoprocarb and cyhalothrin resistance. The expression levels of putative *CarE* genes were examined and compared among IS-R, CY-R, and SS strains, and only the *R. padi* carboxylesterase gene (*RpCarE*) was significantly over expressed in both the IS-R and CY-R strains compared to the SS strain. The coding region of the *Rp*CarE gene was cloned and expressed in *Escherichia coli*. The purified RpCarE protein was able to catalyze the model substrate, α-naphtyl acetate (*K*cat = 5.50 s^-1^; *K*m = 42.98 μM). HPLC assay showed that the recombinant protein had hydrolase activity against isoprocarb and cyhalothrin. The modeling and docking analyses consistently indicated these two insecticide molecules fit snugly into the catalytic pocket of RpCarE. Taken together, these findings suggest that RpCarE plays an important role in metabolic resistance to carbamates and pyrethroids in *R. padi*.

## Introduction

The bird cherry-oat aphid, *Rhopalosiphum padi*, is a serious worldwide wheat pest. *R. padi* causes serious damage to wheat by directing extracting nourishment and transmitting barley yellow dwarf virus (Du et al., 2007; Wang et al., 2016). In China, aphid control is dependent primarily on the application of chemical insecticides, including carbamates, and pyrethroids. Unfortunately, the extensive application of insecticides has resulted in the development of resistance in field populations of *R. padi* (Chen et al., 2007; Zuo et al., 2016). Thus, uncovering the resistance mechanism and its molecular basis is essential to better control this important pest.

The two main mechanisms predominantly responsible for insecticide resistance are target site insensitivity and metabolic resistance due to elevated levels of insecticide detoxifying enzymes (Li et al., 2007; Liu, 2015; Barres et al., 2016). Biochemical assays have demonstrated the resistance to carbamates and organophosphates caused by insensitive acetylcholinesterase (ACE) in a lot of insect species (Hemingway et al., 2004; Bass et al., 2014). A S431F substitution of ACE-1 in *Myzus persicae* resistant clones was correlated with the resistance of the species to pirimicarb (Nabeshima et al., 2003). The A302S in ACE-1 was found to be associated with organophosphates resistance in *Aphis gossypii* (Li and Han, 2004). The substitutions in ACE disturbed both the space and hydrophobicity, thus, preventing pirimicarb from interacting with the active site in the resistant *M. persicae* and *A. gossypii* (Nabeshima et al., 2003; Andrews et al., 2004). Mutations in the voltage-gated sodium channel, a trans-membrane ion channel that plays an essential role in the initiation and propagation of action potentials in neurons, are involved in resistance to pyrethroids (Zlotkin, 1999). L1014F and M918T initially identified in the housefly and, respectively, referred as *kdr* and *super-kdr* mutations are two most common mutations in the voltage-gated sodium channel (Williamson et al., 1996; Franck et al., 2012). The *kdr* mutation can cause moderate resistance to DTT and pyrethroids, whereas the *super-kdr* mutation is usually linked to the *kdr* and shown to significantly enhance the resistance phenotypic expression due to *kdr* (Vais et al., 2000; Eleftherianos et al., 2008; Franck et al., 2012). The *kdr* or *super-kdr* mutations have been indentified in the pyrethroid resistant strains of several aphid species (Eleftherianos et al., 2008; Marshall et al., 2012; Foster et al., 2014). The metabolic resistance has evolved by the amplification, overexpression and coding sequence variation of three major detoxification enzymes, i.e., cytochrome P450 monooxygenases (P450s), carboxylesterases (CarE), and glutathione-S-transferases (GSTs) (Hemingway and Ranson, 2000; Hemingway et al., 2004; Li et al., 2007; Ramsey et al., 2010).

In recent years, *R. padi* had developed resistance to some insecticides (Zuo et al., 2016; Zhang et al., 2017). Regional susceptibility analyses of 12 *R. padi* field populations has shown that the populations varied in their resistance levels to the tested insecticides, with the highest resistant ratio of 13.6, 18.2, 13.1, and 12.1 to imidacloprid, bifenthrin, decamethrin, and abamectin, respectively (Zuo et al., 2016). Both insecticide target site mutations and metabolic resistance were found in this aphid. Acetylcholinesterase gene mutations might contribute to the resistance of this pest to organophosphate and carbamate insecticides (Chen et al., 2007; Bettaibi et al., 2016). Significant higher GST activity was found in two field populations collected in China (Zhang et al., 2017). In the imidacloprid resistant strain of *R. padi*, the *CYP6CY3-1* and *CYP6CY3-2* were significantly overexpressed (Wang et al., 2018). So far, information about CarE from *R. padi* and its role in insecticide resistance is still unavailable.

Carboxylesterases (CarEs, EC 3.1.1.1) are a superfamily of metabolic enzymes that hydrolyse carboxylic ester bonds with the addition of water and are thought to play important physiological roles in xenobiotic metabolism (Campbell et al., 2003; Wheelock et al., 2005; Birner-Gruenberger et al., 2012). The overexpression of CarEs has been associated with carbamate and pyrethroid resistance in several insects (Wheelock et al., 2005). Carboxylesterase E4, which is produced by resistant *M. persicae,* both hydrolyses and sequesters the insecticide, leading to carbamate and pyrethriod resistance in these aphids (Devonshire and Moores, 1982; Lan et al., 2005). Elevated esterase hydrolysis activity is related to cyhalothrin resistance in *Aphis glycines* (Xi et al., 2015).

In the present work, isoprocarb-resistant (IS-R) and cyhalothrin-resistant (CY-R) strains and a relatively susceptible (SS) strain were obtained from the same field population by successive selection with or without insecticide. CarE activity was evaluated among the IS-R, CY-R, and SS strains, and the expression patterns of seven putative CarE or CarE-likes genes were studied by RT-PCR, with the target gene (*RpCarE*) being confirmed and cloned. We heterologously expressed *RpCarE* in *Escherichia coli* cells and purified the fusion protein. Moreover, we measured the activity of the fusion protein against the standard substrate (α-naphthyl acetate) and examined the hydrolase activity against isoprocarb and cyhalothrin. Homology modeling and insecticide docking studies were also conducted to interpret the substrate metabolic detoxification. The current results will contribute to understanding of the mechanism of insecticide resistance mediated by RpCarE.

## Materials and Methods

### Insects

The susceptible strain (SS) originated from a field *R. padi* population collected in 2013 from Gansu Province, China. The isoprocarb resistant strain (IS-R) and cyhalothrin resistant strain (CY-R) were successively selected by exposing isoprocarb or cyhalothrin for more than 60 generations. The IS-R strain showed an LC_50_ of 33.436 mg L^-1^ for isoprocarb, with an ∼32.4-fold increased resistance compared with the SS (LC_50_ of 1.032 mg L^-1^ for isoprocarb). The CY-R strain (LC_50_ of 8.858 mg L^-1^ for cyhalothrin) displayed ∼27.8-fold increased resistance compared to the SS (LC_50_ of 0.319 mg L^-1^ for cyhalothrin) (Supplementary Table S1). During the insecticide selection, the toxicity of insecticides was evaluated every four generations, and the evaluated LC_50_ values were used as the selection concentration for the following four generations. Resistance ratio = LC_50_ of resistant strain/LC_50_ of susceptible strain. All insects were reared on seedlings of wheat (cultivar “Xiaoyan 22”) in mesh cages (41 cm × 41 cm × 41 cm) in the laboratory at 23 ± 1°C, 70% relative humidity and a photoperiod of L16:D8.

### Chemicals

The insecticides used for bioassays included isoprocarb (95% purity, Anhui Huaxing Chemical industry Co. Ltd., China) and cyhalothrin (96% purity, Yancheng Nongbo Bio-technology Co. Ltd., China).

α-naphthol (α-N) and fast blue RR salt were products of Sinopharm Chemical Reagent Co. Ltd. (Shanghai, China). α-naphthyl acetate (α-NA) was purchased from Solarbio (Beijing, China). All other chemicals were of analytical grade and purchased from commercial suppliers.

### Carboxylesterase Activity Assays

Carboxylesterase (CarE) activity was determined by the method of Han et al. (2012) and Li et al. (2016) with modification. All aphids tested were fed on seedlings (three-leaf stage) of wheat cultivar “Xiaoyan 22” under the aforementioned condition, and did not contact with any insecticides from the first instar to the adult before enzyme activity assays. Ten 9-days old apterous adult specimens were taken from each of the three strains (IS-R, CY-R, and SS), homogenized on ice in 1 mL of pre-chilled PBS (0.1 mol L^-1^, pH 7.5, containing 1.0 × 10^-3^ mol L^-1^ EDTA) and centrifuged at 4°C and 12,000 × *g* for 10 min. The enzyme source was supernatant of the homegenized specimens. Supernatants were used for testing. Protein concentrations were determined using the Bradford method with bovine serum albumin as the standard (Bradford, 1976). The assay mixture contained 100 μL of substrate solution (10 mM α-NA and 3 mM Fast Blue RR salt, pH 6.0) and 100 μL of enzyme solution. After incubation at 30°C for 10 min, assays were conducted at 30°C in 96-well microplates, and absorbance was measured at 450 nm in a microplate reader (M200 PRO, Tecan, Männedorf, Switzerland). The experiment was repeated three times.

### Screening of *R. padi* CarE Genes Associated With Insecticide Resistance

Putative CarE genes were systematically searched based on *R. padi* transcriptome data (Duan et al., 2017) and re-sequenced via PCR amplification. Sequences of the seven CarE genes obtained were deposited in GenBank database. The GenBank accession numbers for each sequence are: CL2012, MH561903; CL3077, MH561904; CL869, MH561905; U11937, MH561906; U14486, MH561907; U4474, MH561908; and U6896, MH561909. Ten apterous adult aphids from each of the three strains (IS-R, CY-R, and SS) were subjected to total RNA extraction using Trizol (Invitrogen, Carlsbad, CA, United States) and Direct-zol RNA miniprep kit (Zymo, Irvine, CA, United States) according to the manufacturer’s protocol, including a DNAse treatment. First-strand cDNA was synthesized using the M-MLV reverse transcriptase cDNA Synthesis Kit (Promega, Madison, WI, United States). RT-qPCR was performed in a final volume of 20 μL, including 10 μL of FastStart Essential DNA Green Master (Roche, NJ, United States), 0.8 μL of each specific primer (**Table 1**), 2 μL of cDNA template, and 6.4 μL of RNase-free water. The reaction was performed with the thermal cycler program: 95°C for 10 min, followed by 40 cycles of 95°C for 10 s, 58°C for 20 s, and 72°C for 20 s. A melting curve was determined (ramping from 55°C to 95°C by 0.5°C every 5 s) to confirm the amplification of specific PCR products. The *R. padi β-actin* and *EF-1α* (elongation factor 1α) genes were used as the internal control (**Table 1**). RT-qPCR was performed on a LightCycler Nano System (Roche, Mannheim, Germany), and the relative expression level was calculated using the 2^-ΔΔCt^ method (Pfaffl, 2001). qPCR experiments were repeated using three biological replicates using aphids from the same generation, and each replicate was performed at least three times.

**Table 1:** Primers used for qRT-PCR, RACE, cloning and protein expression.

Primer application	Primer name	Sequence (5′ – 3′)
qRT-PCR	CL2012F	TGAAAATCACAGAGTCGCAGCC
	CL2012R	CAGAAAACCATTGTCGTCCTTG
	CL3077F	AATCGGGAAGCACAGACG
	CL3077R	TAGACCTACTGTTGCCCCA
	CL869F	TAAGCACCGAAGACGACG
	CL869R	GAAACAGCCCAACGACAC
	U11937F	AAGTAGTCGGAAGTGAAGATTG
	U11937R	AAAGGTAGTGGGGACCATAGCC
	U14486F	GGATGTTTGACAGGAGGCTT
	U14486R	CAAGGACTACACTACAAAACACGA
	U4474F	CGGCATCGGATACGCCTAAAG
	U4474R	TCCAAACCAAGGCTTTACGGG
	U6896F	GTTCCACGAAAGAAAATGACTG
	U6896R	TTGACAGGAGGCTTGAATCT
	*β*-actinF	TGAGACATTCAACACCCCTG
	*β*-actinR	CCTTCATAGATTGGGACAGTG
	*qEF-1αF*	GCTCTATTGGCTTTCACCTT
	*qEF-1αR*	GATGTAACTGCTGACTTCTTTC
RACE	*RpCarE* -3R1	CTGGATTACAAGTTCGTCCCATCTA
	*RpCarE* -3R2	GTTCCGTAATAATCTGTCTGGAGTC
Cloning full coding region	*RpCarE* -CF	CAGTGCGTCCTGGGCCGTAATCT
	*RpCarE* -CR	GTTTTCAATCAATGTTATGTGGG
Protein expression	*Rp*CarE-BamHI	CGCGGATCCATGGAAGTGGTCATCGAACAAGGT;
	*Rp*CarE-HindIII	CCCAAGCTTTTAAACAATGGATTCTTTTATTAA


### Cloning of Carboxylesterase From *R. padi*

Based on the above-mentioned screening of CarE genes from *R. padi* transcriptome data (Duan et al., 2017), a carboxylesterase gene (CL2012, hereinafter referred as *Rp*CarE)) which was over-expreesed in the resistant strains was confirmed by qPCR, and the coding region was cloned by RT-PCR. Pre-analysis showed the 5′ sequences of *RpCarE* from *R. padi* transcriptome were sufficient for protein expression. Gene-specific primes were used for 3′-RACE (*RpCarE*-3R1 and *RpCarE*-3R2) to clone the 3′ sequences of the gene. To confirm the accuracy of the *RpCarE* linked from the 3′-RACE results, a specific primer pair (*RpCarE*-CF and *RpCarE*-CR) was designed to amplify the full coding region of the gene. The primers used for RACE are shown in **Table 1**. The amplification reaction mix contained 2 units of Taq DNA polymerase (5 U/μL, Sangon Biotech Co. Ltd., Shanghai, China), 100 μM dNTPs, 4 mM MgCl_2_, 0.4 μM of forward and reverse primers, and 1 μL of template DNA. All purified PCR products were cloned into pGEM-T easy vectors (Promega, Madison, WI, United States) and transformed into *Escherichia coli* DH5α competent cells (Takara, Kyoto, Japan). To ensure that the correct cDNA sequences were obtained, three positive clones from each sample were randomly chosen for bidirectional sequencing on an Applied Biosystems 3730 automated sequencer (Applied Biosystems, Foster City, CA, United States).

### Sequence Analysis

Sequence identification and similarities were analyzed using BLAST^1^. Amino acid sequences of RpCarE and homologs from other insect species were aligned using ClustalW2 software (Larkin et al., 2007). The molecular weight (MW) and theoretical isoelectric point (pI) of RpCarE were calculated using the ExPASy (Gasteiger et al., 2003). Signal peptides were predicted using the Signal P4.1 software (Petersen et al., 2011).

### Protein Expression/Purification and Western Blot Analysis

The encoding region of *RpCarE* was amplified from the pGEM-T/*Rp*CarE plasmid with gene specific primers (*Rp*CarE -BamHI: CGCGGATCCATGGAAGTGGTCATCGAACAAGGT; *Rp*CarE-HindIII: CCCAAGCTTTTAAACAATGGATTCTTTTATTAA) introducing BamHI and HindIII restriction sites (underlined). Amplicon was purified and digested with corresponding restriction enzymes and subcloned into pET-28a (Novagen, Merck, Germany). The plasmid was verified by restriction digestion and nucleotide sequencing. The expression of *Rp*CarE in the *E. coli* BL-21 (DE-3) strain (Takara, Kyoto, Japan) was induced using Isopropyl β-D-thiogalactoside (IPTG, with final concentration of 0.4 mM). Cultured cells were harvested by centrifugation (6,000 × *g*, 20 min, 4°C). The resulting pellet was re-suspended in 50 mM Tris-HCl (pH 8.0) and lysed by sonication (Sonics Vibra-Cell, 130 w, 30% power). Cell lysate was centrifuged at 12,000 × *g* for 30 min, and the supernatant was incubated with 2 mL of cOmplete His-Tag Purification Resin (Roche) for 1 h with shaking, and protein was eluted using lysis buffer supplemented with 50 mM imidazole. Protein concentrations were determined using the Bradford method with bovine serum albumin as the standard (Bradford, 1976).

Recombinant RpCarE was analyzed using 12% SDS-PAGE and stained with Coomassie Brilliant Blue R-250. For western blot, separated proteins were electrotransferred to a PVDF membrane (Millipore), and the membrane was subsequently blocked with 5% skim milk powder in TBST for 1 h. Target proteins were verified with mouse anti-His mouse monoclonal antibodies (CWBIO) followed by staining with goat-anti-mouse IgG (CWBIO). Blots were visualized using WesternBright ECL kit (Advasta), and signal was detected using a chemiluminescence imaging system (Clinx Science Instruments, Shanghai, China).

### Determination of Enzymatic Activity

The kinetics of purified RpCarE protein against α-NA was determined using the method of Li et al. (2016) with modification. Reactions were carried out in a 96-well microplate, with each well containing 100 μL of appropriately diluted purified protein in Tris-HCl buffer and 100 μL of α-NA in buffer (3 mM Fast Blue RR salt and increasing concentrations of α-NA). The formation of α-N was recorded at 450 nm for 5 min in a microplate reader (M200 PRO, Tecan, Männedorf, Switzerland) and quantified using α-N standard curves. The values for *k*cat and *K*m were estimated using the “Hyper32” hyperbolic regression software (Xie et al., 2014).

### HPLC Analysis of Insecticide Metabolism

Assays for the hydrolysis of isoprocarb and cyhalothrin were carried out by monitoring substrate loss with a Hitachi D-2000 Elite HPLC system (Hitachi High-Technologies Corporation, Tokyo, Japan) as described by Li et al. (2016), with some modifications. First, 100 μL of isoprocarb and cyhalothrin (each in 100 μM) was incubated separately with 100 μL of recombinant RpCarE protein (0.2 mg mL^-1^). Insecticides with heat-inactivated enzyme served as controls. Incubation reactions were carried out at 30°C for 1 h and stopped by addition of 100 μL acetonitrile. Samples were centrifuged at 12,000 × *g* for 10 min before transferring the supernatant to HPLC vials. Ten microliter of the supernatant were injected onto a reverse-phase Symmetry C18 column (250 mm × 4.6 mm, 5 μm, Waters Crop., Milford, MA, United States) with a flow rate of 1 mL min^-1^ at 30°C for 30 min. Reactions were run with a mobile phase (80% acetonitrile: 20% water) and monitored at 215 and 230 nm for isoprocarb and cyhalothrin, respectively.

### *Rp*CarE Modeling and Substrate Docking

The molecular model of *Rp*CarE was created as described by Zhang (2008) and Elzaki et al. (2017) using the I-TASSER on-line server^2^. Isoprocarb and cyhalothrin molecules were docked into the active site of *Rp*CarE using the Surflex-Dock (SFXC) function in the SYBYLx2.0 software (Tripos, St. Louis, MO, United States). Final figures were prepared using the PyMOL program (DeLano, 2002).

### Statistical Analysis

The significance of the differences in mRNA levels and enzyme activities was determined by non-parametric Mann–Whitney *U-*test with the level of significance at *P* < 0.05. Data analyses were performed using SPSS Version 21.0 software (SPSS Inc., Chicago, IL, United States).

## Results

### Determination of CarE Activity

**FIGURE 1:**
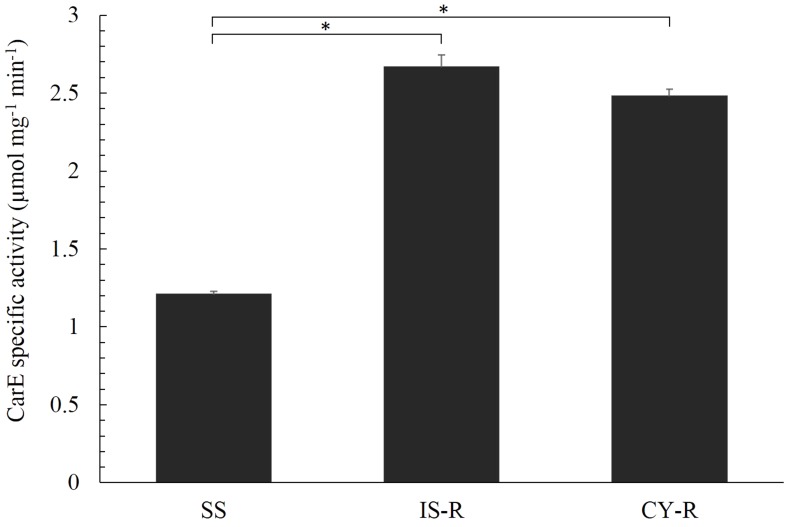
Specific activity of *R. padi* carboxylesterase for α-NA in IS-R, CY-R, and SS strains. The results of three replicates were averaged from three different protein preparations. Vertical bars represent the standard error of the mean from three independent replicates. Asterisk indicates significant differences by Mann–Whitney *U*-test (*P* < 0.05).

Carboxylesterase activities of different strains were determined using α-NA as a substrate. IS-R and CY-R strains exhibited significantly higher carboxylesterase activity (2.20- and 2.05-fold) compared with the susceptible strain (*P* < 0.05) (**Figure 1** and Supplementary Table S2).

### CarE Gene mRNA Level Determination

**FIGURE 2:**
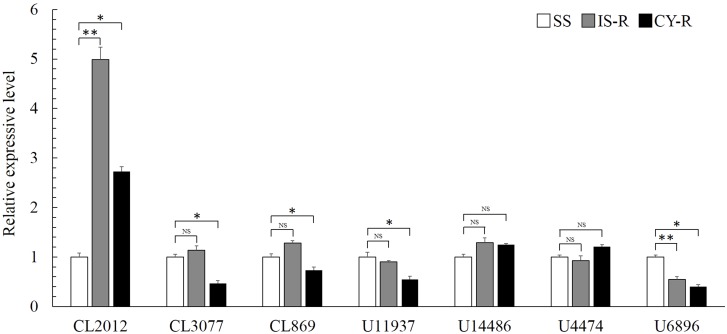
Comparison of carboxylesterase gene mRNA levels among the isoprocarb resistant (IS-R), cyhalothrin resistant (CY-R), and susceptible (SS) strains. Values are plotted as the mean ± SE of three repeats. Vertical bars represent the standard error of the mean of three independent replicates. Asterisk indicates significant differences by Mann–Whitney *U*-test (^∗^*P* < 0.05, ^∗∗^*P* < 0.01).

Seven putative carboxylesterase genes were obtained from the transcriptome database of *R. padi* (**Table 1**), and compared among susceptible and resistant strains. Relative expression levels of CL2102 were 4.99- and 2.73-fold higher in IS-R and CY-R than in the susceptible strain, respectively (**Figure 2**). Relative expression analysis of the seven carboxylesterase genes revealed that CL2102 was moderately abundant in the isoprocarb resistant and cyhalothrin resistant strains of *R. padi*, whereas the other six carboxylesterase genes was lowly abundant in the two resistant strains.

### cDNA Amplification

**FIGURE 3:**
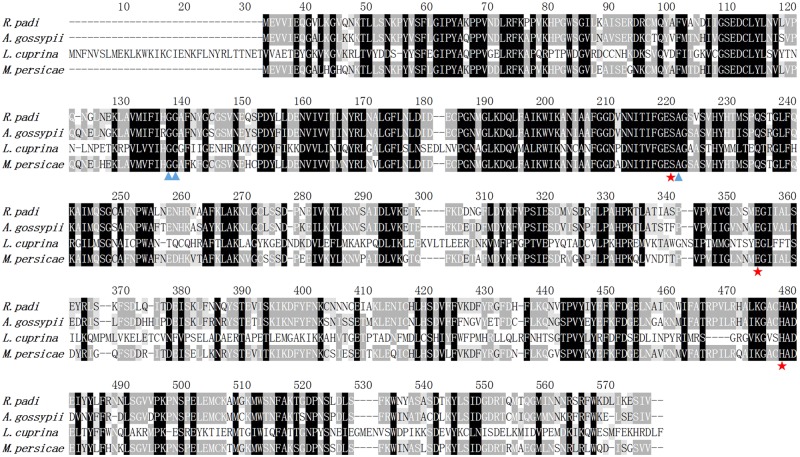
Alignment of the deduced amino acid sequences of *R. padi* carboxylesterase with the previously reported carboxylesterases from *A. gossypii* (GenBank accession number XP_022165140), E3 from *Lucilia cuprina* (GenBank accession number XP_022165140), and FE4-like from *M. persicae* (GenBank accession number XP_022165140).

To obtain the complete sequence, gene-specific primers for CL2102 were designed for amplification of the 3′ cDNA ends. The resulting carboxylesterase cDNA sequence contains an open reading frame (ORF) of 1,581 bp that encodes a putative protein containing 526 amino acid residues. Its calculated molecular weight is 59.22 kDa, and the predicted isoelectric point is 6.61. No signal peptide was detected upon amino acid sequencing. Homology analysis against the already published genes in GenBank indicated that carboxylesterase shares high identity with *M. persicae* FE4-like esterase (XP_022165140; 80% identity), *Acyrthosiphon pisum* FE4 esterase (XP_001951456; 80% identity), *A. glycines* carboxylesterase (AEI70326; 78% identity), and *A. gossypii* carboxylesterase (BAE66715; 78% identity). The alignment shows that this *R. padi* carboxylesterase has highly conserved residues that form an atalytic triad (S185-H432-E312) (**Figure 3**).

### Expression of *Rp*CarE in *E. coli* and Verification via Western Blot

**FIGURE 4:**
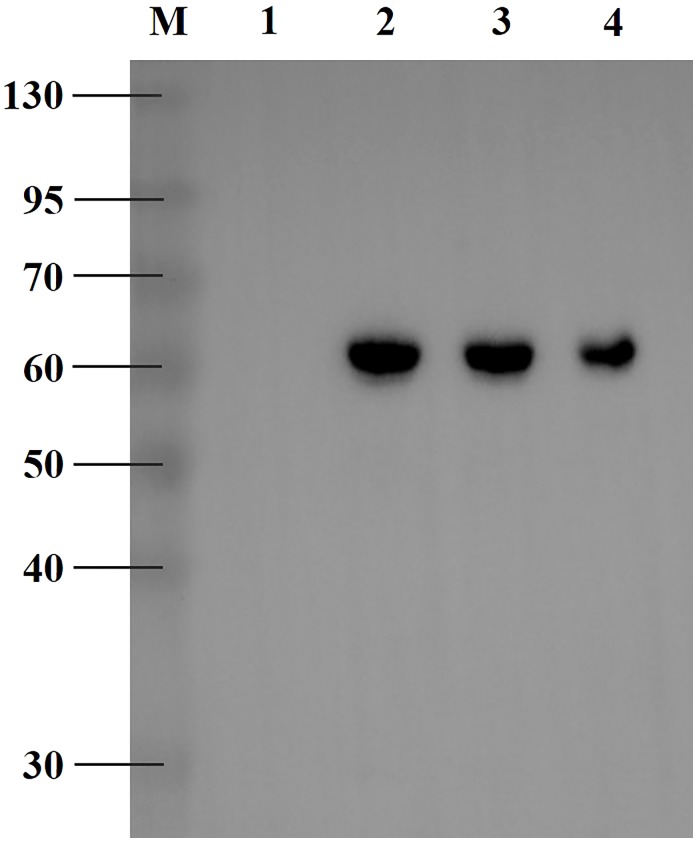
Western blot analysis of exogenously expressed *Rp*CarE. The RpCarE protein was verified via His-mouse antibody. M, protein molecular markers; line 1, total proteins from uninduced *E. coli* cells; line 2, total proteins from induced *E. coli* cells; line 3, soluble proteins from induced *E. coli* cells; line 4, purified fusion protein.

To functionally express *Rp*CarE, its coding sequence was inserted into the pET-28a expression vector and expressed in the BL-21 (DE-3) *E. coli* strain. Cells harvested 12 h after IPTG induction were considered to be the most suitable for protein expression and isolation based on the Coomassie blue-stained sodium dodecyl sulfate polyacrylamide gel electrophoresis (SDS-PAGE) images (data not shown). Immunoreactivity of the target proteins for His-mouse antibodies in the western blot are shown in **Figure 4**. The fusion protein with a His6 tag migrated as a single band with a molecular mass of ∼60 kDa, which matched the calculated molecular mass of 59.22 kDa. These results indicate that *Rp*CarE was successfully expressed in *E. coli*.

### *Rp*CarE Activity for α-Naphthyl Acetate

The activity of the purified recombinant enzyme toward α-NA is shown in **Table 2**. *Rp*CarE exhibited high catalytic efficiency with a *K*m of 42.98 μM, a *K*cat of 5.50 s^-1^ and a specific activity of 4.43 μM s^-1^(μM^-1^⋅protein). These results suggest that the purified recombinant protein (RpCarE) has a high affinity and turnover of substrate.

**Table 2:** Kinetic parameters for the purified *R. padi* carboxylesterase toward the α-naphthyl acetate.

Enzyme	α-Naphthyl acetate				
	
	*V*_max_	*K*_m_	*K*_cat_	*K*_cat_/*K*_m_	Specific activity
*Rp*CarE	1.18 ± 0.66	42.98 ± 6.73	5.50 ± 0.28	0.13 ± 0.02	4.43 ± 0.11


*Kcat, catalytic constant; Km, Michaelis constant; Vmax, Maximum Velocity. Units: Km: μM; Kcat: s^-1^ ; Kcat/Km: 1/(s × μM). The results are shown as the mean ± SE (n = 3).*

### *Rp*CarE Metabolism of Isoprocarb and Cyhalothrin

**FIGURE 5:**
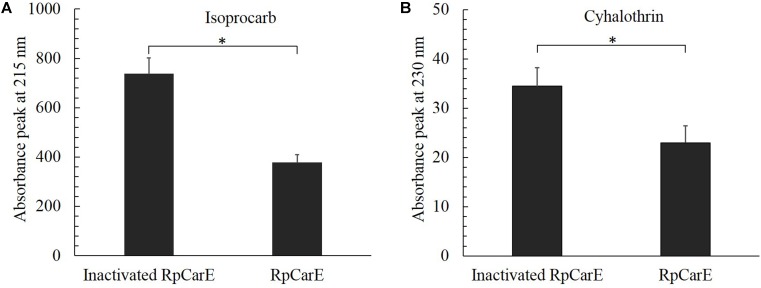
Hydrolytic activity of purified fusion *Rp*CarE protein against isoprocarb **(A)** and cyhalothrin **(B)**. Insecticide metabolism was analyzed using a Hitachi D-2000 Elite HPLC system. Isoprocarb and cyhalothrin were monitored via their absorption at 215 and 230 nm, respectively, and quantified via their peak integration. A control was prepared using heat-inactivated *Rp*CarE. Values are shown as the mean ± SE of three repeats. Vertical bars represent the standard error of the mean of three independent replicates. Asterisk indicates significant differences by Mann–Whitney *U*-test (*P* < 0.05).

Specific activities of the purified recombinant enzyme against isoprocarb and cyhalothrin were assessed by measuring substrate depletion. As shown in **Figure 5**, the fusion protein exhibited significant activity toward both isoprocarb and cyhalothrin.

### The Binding Mode of Insecticides to *Rp*CarE

**FIGURE 6:**
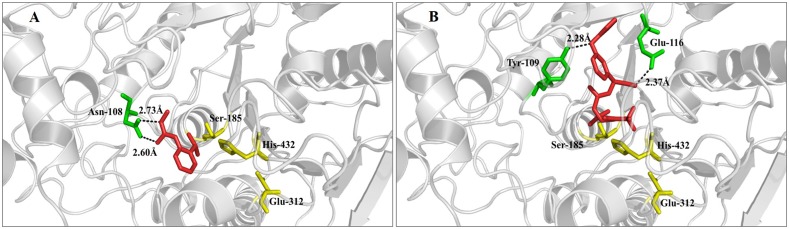
Binding models of the complex formed by *Rp*CarE and insecticides. **(A)** Isoprocarb-*Rp*CarE, **(B)** cyhalothrin-*Rp*CarE. Insecticides are displayed as red sticks. Residues of the catalytic triad (serine, glutamate, and histidine) are represented as yellow sticks. Other residues that interact with the substrate are shaded green.

To better understand the underlying mechanism causing *Rp*CarE to metabolize insecticides, a molecular docking simulation was conducted using the homology mode of *Rp*CarE with insecticides, including isoprocarb and cyhalothrin. The docking experiments were conducted using the Surflex-Dock (SFXC) program from the SYBYL X2.0 software. The RpCarE protein model was generated based on the crystal structure of *Lucilia cuprina* α*-E7*. *Rp*CarE contains several conserved CarE characteristics, such as the canonical catalytic triad (serine, glutamate, and histidine) and the oxyanion hole (alanine and glycine) (**Figure 3**). Modeling and docking analyses showed that isoprocarb and cyhalothrin fit snugly into the catalytic pocket of *Rp*CarE (**Figure 6**). The Asn-108 residue was predicted to anchor isoprocarb by hydrogen donors (**Figure 6A**). Tyr-109 and Glu-116 were the major determinants in cyhalothrin binding, positioning the molecule close to catalytic triads (Ser185-His432-Glu312) (**Figure 6B**). These results indicate that the active site pocket of *Rp*CarE is ideally shaped for isoprocarb and cyhalothrin and is able to effectively metabolize these insecticides.

## Discussion

Carboxylesterases, an important detoxification enzyme family, are involved in mediating the metabolic resistance to carbamate, organophosphate and pyrethroid insecticides in many insect species (Wheelock et al., 2005). To reveal the CarE-mediated metabolism of isoprocarb and cyhalothrin in *R. padi*, one isoprocarb resistance strain (IS-R), and one cyhalothrin resistance strain (CY-R) were obtained by successive selection with insecticides. CarE activities were measured in IS-R, CY-R and susceptible strains of *R. padi*, yielding significantly higher activities in the IS-R and CY-R strains than in the SS strain (**Figure 1**), suggesting a correlation between CarE and isoprocarb and cyhalothrin resistance. Furthermore, the hydrolytic activity of a *R. padi* CarE (*Rp*CarE) to isoprocarb and cyhalothrin suggest that *Rp*CarE is involved in resistance to the chemicals. In the early 1970s, resistant strains of *M. persicae* were shown to possess improved hydrolytic activity against the esterase standard substrate α-NA (Needham and Sawicki, 1971). Further study revealed that EF4 accounts for the broad spectrum of resistance to carbamate and pyrethroid insecticides (Devonshire and Moores, 1982). CarE activity in *A. gossypii* was determined using several standard substrates and was shown to be significantly higher in omethoate, deltamethrin, and malathion resistant strains than in susceptible strains (Cao et al., 2008a,b; Pan et al., 2009). There was a significant difference in carboxylesterase activity between a beta-cyperthrin-resistant strain (4,419-fold) and a susceptible strain of *Musca domestica* (Zhang et al., 2007).

Based on sequence alignment and conserved motifs, seven carboxylesterase and carboxylesterase-like genes were identified. The relative expression patterns of candidate genes were determined in the insecticide resistant and susceptible strains. Among the seven CarE genes, only CL2012 (*RpCarE*) was found to have significantly increased expression levels in both the IS-R and CY-R compared to the SS. A homology search of CL2012 showed maximum similarity (86%) to *A. gossypii* carboxylesterase. Therefore, we proposed that the CL2012 gene, as the carboxylesterase gene of *R. padi*, might participate in isoprocarb and cyhalothrin resistance. Twenty-eight CarE and CarE-like genes were identified in the transcriptome of *Laodelphax striatellus*, and *LsCarE1* was significantly overexpressed in the chlorpyifos-resistant and chlorpyifos-relaxed selection strains by 32.06- and 8.6-fold, respectively. Additionally, overexpressed *LsCarE1* mediated chlorpyifos resistance in *L. striatellus* (Zhang et al., 2012). In the deltamethrin-resistant strain of *A. gossypii*, the transcript levels of carboxylesterase were significantly enhanced, and this up-regulation was responsible for the development of resistance to deltamethrin (Cao et al., 2008a). The enhanced activity of carboxylesterases of *M. persicae* confers broad spectrum resistance to organophosphate, carbamate and pyrethroid (Bass et al., 2014). The *M. persicae* field population resistant to methomyl and omethoate exhibited a higher carboxylesterases activity compared to the laboratory susceptible strain (Tang et al., 2017). Three populations of the cowpea aphid (*Aphis craccivora*) collected in Egypt showed higher carboxylesterases which may cause the resistance of the species to organophosphates, carbamates, and neonicotinoids (Fouad et al., 2016). CarE enzyme activity were positively correlated with resistance level to chlorpyrifos, deltamethrin, and methomyl in the field populations of *Sitobion avenae* collect in wheat fields (Zhang et al., 2017).

There are a conserved catalytic triad (Ser, His, and Glu) and an oxyanion hole for carboxylesterases (Stok et al., 2004; Wheelock et al., 2005). During hydrolysis of carboxylesterases, a nucleophilic attack occurs by Ser on the carbon of the carbonyl group, which is next transferred to the His from the catalytic Ser. The protonated His is, in turn, stabilized via a hydrogen bond to the Glu (Stok et al., 2004). The Ser nucleophile attacks the substrate and forms the first tetrahedral intermediate, which is stabilized by two Gly residues in the oxyanion hole (Wheelock et al., 2005). The intermediate rapidly collapses to produce the acyl-enzyme complex, which then undergoes attacks by an His-activated water molecule and forms the second tetrahedral intermediate. The acid component of the substrate is released after the rapid rearrangement (Stok et al., 2004; Wheelock et al., 2005). To further investigate the role of *RpCarE* in resistance to isoprocarb and cyhalothrin in *R. padi*, the full coding region was cloned and characterized. The deduced amino acid sequence of *Rp*CarE was found to exhibit striking homology to CarEs in other insects, including the highly conserved catalytic triad (Ser, His, and Glu) and an oxyanion hole consisting of backbone amide group of Ala, Gly, and Gly which indicate that *Rp*CarE can function as an active esterase (Tsubota and Shiotsuki, 2010; Jackson et al., 2013).

*Rp*CarE was cloned into pET-28a and expressed the in *E. coli*. The molecular mass was similar to the carboxylesterase E4 in *M. persicae* (60 kDa) (Lan et al., 2005) and differed from that of *A. gossypii* (65 kDa) (Gong et al., 2017). The purified fusion protein displayed significant hydrolase activity against the model substrate α-naphthyl acetate with a *K*cat of 5.50 s^-1^, suggesting *Rp*CarE was active and successfully expressed in the *E. coli* strain. Fusion proteins of carboxylesterase 001D from *Helicoverpa armigera* expressed in *E. coli* showed enzyme activities against α-NA with a *K*cat between 0.35 and 2.29 s^-1^ (Li et al., 2016). HPLC showed that purified RpCarE can metabolize the two insecticide substrates, isoprocarb and cyhalothrin, *in vitro*, indicating that the carboxylesterase is involved in the detoxification of isoprocarb and cyhalothrin in *R. padi*. Many studies have demonstrated that carboxylesterases can metabolize organophosphorus, carbamate and pyrethroid insecticides in pest insects (Bass and Field, 2011; Coppin et al., 2012; Li et al., 2013). The E4 carboxylesterase degraded 64% of carbaryl and 80% of malathion within 2.5 and 1.25 h, respectively (Lan et al., 2005). All recombinant expressed wild type and mutant *A. gossypii* carboxylesterases can hydrolyse paraoxon and parathion to varying degrees (Gong et al., 2017). Li et al. (2016) showed that the *H. armigera* carboxylesterase 001D exhibited low but measurable hydrolase activity toward *β*-cypermethrin and fenvalerate. Fifty percent of malathion and 89% of malathion were hydrolysed by recombinant D1CarE5 within 25 and 100 min, respectively, (Xie et al., 2013).

Our modeling and docking data were in accordance with our metabolism analyses. Some residues created a hydrophobic interface at the binding cavity, and critical residues anchored the insecticide molecule. In our study, isoprocarb and cyhalothrin were docked into the predicted active site pocket and were further stabilized by hydrogen bonds with Asn-108, Tyr-109, and Glu-116. These analyses predicted that *Rp*CarE is capable of hydrolysing isoprocarb and cyhalothrin. *L. cuprina* α*-E* (*Lc*αE7), the template for *Rp*CarE model building, showed a strong hydrophobic binding ability. Molecular modeling of *Lc*αE7 indicated that some mutations in the enzyme were responsible for organophosphates resistance of *L. cuprina* (Yan et al., 2009). *Lc*αE7 could sequester the insecticide molecule and slowly detoxify it; this high affinity gave *Lc*αE7 an important function in insecticide resistance (Jackson et al., 2013). The *A. gossypii* CarE protein models based on the crystal structure of *Lc*αE7 showed that some of non-synonymous mutations (H104R, A128V, T333P, and K484R) affected the active site pocket and binding energy, resulted in different binding affinity between *A. gossypii* CarE with insecticide compounds (Gong et al., 2017).

In summary, we cloned a carboxylesterase gene from *R. padi* and overexpressed it in both isoprocarb-resistant and cyhalothrin-resistant strains. *Rp*CarE was successfully expressed in *E. coli* and exhibited hydrolytic activity toward the model substrate α-NA and to isoprocarb and cyhalothrin. These results suggest that *Rp*CarE is involved in resistance to isoprocarb and cyhalothrin in *R. padi*. Further studies will be required to determine the detailed role of *Rp*CarE as well as to clarify whether other metabolic enzymes (e.g., P450s and GST) are involved in metabolic resistance or whether target site mutations can play a role in the insecticide resistance of *R. padi* resistant strains.

## Author Contributions

KW and MC designed the research and wrote the paper. KW, YH, and XL performed research. KW analyzed data.

## Conflict of Interest Statement

The authors declare that the research was conducted in the absence of any commercial or financial relationships that could be construed as a potential conflict of interest.

## Footnotes

1 blast.ncbi.nlm.nih.gov/blast/

2 https://zhanglab.ccmb.med.umich.edu/I-TASSER/
