# Supplementary material for: Functional Analysis of a Carboxylesterase Gene Associated With Isoprocarb and Cyhalothrin Resistance in Rhopalosiphum padi (L.)
Source: Front Physiol. 2018 Jul 25;9:992. doi: 10.3389/fphys.2018.00992 (PMC6068260; doi:10.3389/fphys.2018.00992)
Supplement: Supplementary file 1 [file Table_1.DOCX]

**Table S1 Susceptibility of SS, IS-R and CY-R to isoprocarb or cyhalothrin**

| Insecticide | Strain | n | Slop ± SE | X2 (df) | LC_50_ (95% confidence limit) (mg L^-1^) | LC_90_ (95% confidence limit) (mg L^-1^) | Resistance ratio |
| --- | --- | --- | --- | --- | --- | --- | --- |
| Isoprocarb | SS | 648 | 1.49 ± 0.15 | 5.76 (3) | 1.032 (0.850-1.234) | 7.461 (5.333-12.039) |  |
|  | IS-R | 662 | 1.69 ± 0.16 | 2.97 (3) | 33.346 (27.301-43.167) | 191.174 (124.418-353.497) | 32.4 |
| Cyhalothrin | SS | 632 | 1.74 ± 0.16 | 1.24 (3) | 0.319 (0.253-0.383) | 1.735 (1.395-2.313) |  |
|  | CY-R | 638 | 1.71 ± 0.16 | 4.17 (3) | 8.858 (7.398-11.050) | 49.927 (33.746-87.129) | 27.8 |
