# Supplementary material for: Functional Analysis of a Carboxylesterase Gene Associated With Isoprocarb and Cyhalothrin Resistance in Rhopalosiphum padi (L.)
Source: Front Physiol. 2018 Jul 25;9:992. doi: 10.3389/fphys.2018.00992 (PMC6068260; doi:10.3389/fphys.2018.00992)
Supplement: Supplementary file 2 [file Table_2.DOCX]

**Table S2 Carboxylesterase activities in SS, IS-R and CY-R of *R. padi***

| Strain | Specific activity of enzyme (µmol mg^-1^ min^-1^) | Ratio |
| --- | --- | --- |
| SS | 1.214 ± 0.0510 b |  |
| IS-R | 2.671 ± 0.1587 a | 2.20 |
| CY-R | 2.483 ± 0.1434 a | 2.05 |

Different letters indicate that the means are significantly different by Student’s *t*-test (*P* < 0.05)
